# Recent Progress in Decarboxylative Oxidative Cross‐Coupling for Biaryl Synthesis

**DOI:** 10.1002/ejoc.201700121

**Published:** 2017-04-26

**Authors:** Gregory J. P. Perry, Igor Larrosa

**Affiliations:** ^1^ School of Chemistry University of Manchester Oxford Road M13 9PL Manchester U.K.

**Keywords:** Decarboxylation, C–H activation, Cross‐coupling, Biaryls, Oxidative Coupling

## Abstract

The beginning of the 21st century has seen tremendous growth in the field of decarboxylative activation. Benzoic acid derivatives are now recognised as atom‐economic alternatives to traditional cross‐coupling partners, and they also benefit from being inexpensive, readily available and shelf‐stable reagents. In this microreview we discuss recent developments in the coupling of benzoic acid derivatives either with an arene or with a second benzoic acid derivative, a process often termed decarboxylative oxidative cross‐coupling. These procedures offer great promise for the development of highly selective and atom‐economic cross‐couplings.

## 1. Introduction

The advent of traditional cross‐coupling reactions – coupling between aryl halides and organometallic reagents – revolutionized thoughts relating to the construction of C–C bonds.^1^ More recent developments in the areas of C–H activation and decarboxylative activation have looked to improve the atom economy of these procedures by using more simple and readily available reagents.^2^, ^3^ The field of C–H activation has provoked considerable interest, because – ideally – it would allow for the construction of complex molecules from the simplest starting materials in an elementary fashion. In reality, this methodology faces the significant challenge of selective activation of one C–H bond in the presence of many others. Decarboxylative activation is a complementary strategy that can overcome some of the inherent difficulties of C–H activation, because the carboxyl group can provide a handle for selective transformations. The ready availability of benzoic acid derivatives also makes these processes comparable to C–H activation in terms of cost and atom efficiency.

### 1.1. Brief History of Decarboxylative Oxidative Coupling

**Scheme 1:**
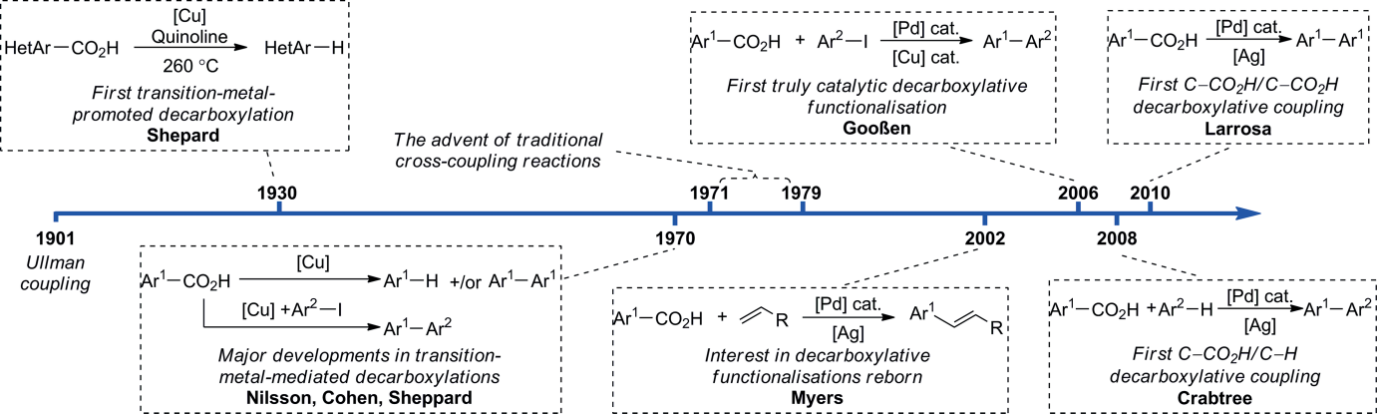
Timeline of significant advancements in decarboxylative oxidative couplings for biaryl synthesis.

The ability of transition metals to promote the decarboxylation of benzoic acid derivatives was first observed in 1930, when Shepard and co‐workers noted that furan‐2‐carboxylic acid derivatives were more prone to protodecarboxylation in the presence of copper than upon heating alone.^4^ Significant advancements were made during the 1970s by the groups of Nilsson,^5^ Sheppard^6^ and Cohen,^7^ who provided a more detailed study of copper‐mediated protodecarboxylation and began demonstrating its generality (Scheme 1). The field remained relatively quiet during the following years, and it was not until 2002 that Myers et al. reported Heck‐type decarboxylative coupling between benzoic acid derivatives and olefins, which – although catalytic in palladium – required a super‐stoichiometric loading of a silver salt additive.^8^ The seminal report in the field of decarboxylative biaryl synthesis was made by Gooßen and co‐workers, who developed direct decarboxylative cross‐coupling between benzoic acid derivatives and aryl iodides.^9^ Most importantly, the group discovered a dimetallic system that used only catalytic amounts of both palladium and copper salts, therefore firmly establishing benzoic acid derivatives as alternative aryl donors in cross‐coupling procedures. In recent years, the coupling of benzoic acid derivatives either with arenes or with other benzoic acid derivatives has attracted much interest. The first reports on C–CO_2_H/C–H and C–CO_2_H/C–CO_2_H couplings were made by the groups of Crabtree^10^ and Larrosa,^11^ respectively. Here we provide a comprehensive review of the current state of decarboxylative oxidative coupling for biaryl synthesis and the successes therein. We also discuss current limitations and the desired goals for the future of this field.

## 2. Protodecarboxylation: Trends in Decarboxylation Aided by Transition Metals

Before discussing decarboxylative oxidative cross‐coupling, we first summarize transition‐metal‐promoted protodecarboxylation. Trends in transition‐metal‐promoted decarboxylation can thus be established, allowing informed prediction on which transition metal is operative in the decarboxylation step of the coupling process. An extensive summary of protodecarboxylation is not the aim of this section, and we only discuss representative procedures. In addition, we restrict our discussion to copper‐, palladium‐ and silver‐catalysed protodecarboxylation, because these metals have been used for decarboxylative cross‐coupling. Other metals, such as gold^12^ and rhodium,^13^ can also promote protodecarboxylation, but we have not included these examples because of their current absence in decarboxylative coupling procedures.

### 2.1. [Cu] Systems

**Scheme 2:**
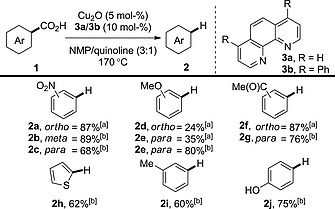
Copper‐catalysed protodecarboxylation of aromatic acids. [a] **3a** (10 mol‐%). [b] **3b** (10 mol‐%).

We have already mentioned that copper was one of the earliest metals found to promote decarboxylation (Section 1.1); however, it was not until the report of a decarboxylative arylation by Gooßen et al. that copper‐catalysed decarboxylation was fully recognised. To assess copper's ability to effect decarboxylative transformations further, the Gooßen group subsequently developed a copper‐catalysed protodecarboxylation procedure (Scheme 2).^14^ This study revealed that benzoic acid derivatives bearing electron‐withdrawing substituents and/or *ortho* substituents were particularly susceptible to decarboxylation. Electron‐rich substrates were generally less reactive than electron‐deficient substrates (compound **2a** vs. **2d**). Also, non‐*ortho*‐substituted benzoic acid derivatives were less reactive than *ortho*‐substituted benzoic acid derivatives; however, good reactivity was still achieved with use of a modified phenanthroline ligand (see **2e**). Copper‐mediated decarboxylation is more general than other transition‐metal‐catalysed procedures, because the latter are limited to acids bearing *ortho* substituents. The main disadvantage is that high temperatures (>170 °C) are required to effect decarboxylation.

### 2.2. [Pd] Systems

**Scheme 3:**
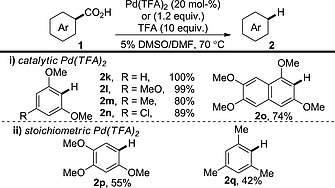
Palladium‐catalysed/promoted protodecarboxylation of aromatic acids.

Investigations by Kozlowski and co‐workers led to a method for palladium‐catalysed protodecarboxylation (Scheme 3).^15^ This procedure represents one of the lowest‐temperature transition‐metal‐catalysed protodecarboxylations to date; however, the scope is severely limited to highly electron‐rich benzoic acid derivatives bearing two *ortho* substituents (Scheme 3, i).^16^ The procedure can be extended to mono‐*ortho*‐substituted and less electron‐rich benzoic acid derivatives, but a stoichiometric amount of palladium is then required (Scheme 3, ii).

Larrosa and co‐workers have also shown that Pd can mediate the protodecarboxylation of biarylcarboxylic acids in the context of a tandem process.^17^

### 2.3. [Ag] Systems

**Scheme 4:**
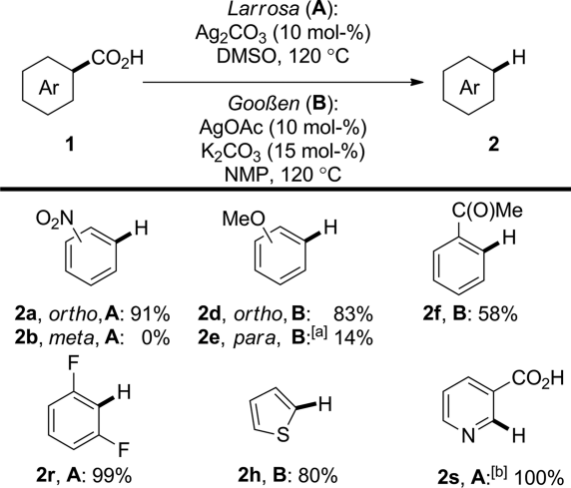
Silver‐catalysed protodecarboxylation of aromatic acids. [a] 160 °C. [b] AcOH (5 mol‐%).

In 2009 the groups of Gooßen^18^ and Larrosa^19^ concomitantly revealed methods for silver‐catalysed protodecarboxylation (Scheme 4).^20^ The decarboxylation is applicable to both electron‐rich and electron‐deficient benzoic acid derivatives (e.g., **2a**, **2d**); however, an *ortho* group is mandatory (**2d** vs. **2e**). The dependence on *ortho* substitution is the key limitation of this procedure over copper‐catalysed processes; however, the decarboxylation of *ortho*‐substituted substrates occurs at somewhat lower temperatures when silver is used (120 vs. 170 °C).

**Scheme 5:**
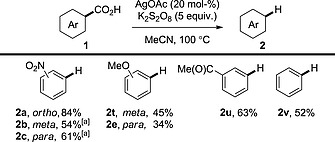
Silver‐catalysed protodecarboxylation of aromatic acids under radical conditions. [a] AgOAc (40 mol‐%).

**Scheme 6:**
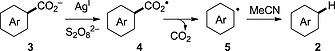
Proposed mechanism for the silver‐catalysed protodecarboxylation of aromatic acids under radical conditions.

A unique system for decarboxylation was reported by Greaney and co‐workers (Scheme 5).^21^ This system employs a silver catalyst, but – because the process takes place in the presence of a strong one‐electron oxidant – a radical pathway for the decarboxylation was proposed (Scheme 6). In this manner, the silver(I) catalyst in combination with K_2_S_2_O_8_ can provide the carboxyl radical **4** from carboxylate **3**. A radical decarboxylation of **4** expels CO_2_ and forms the aryl radical **5**, which can then abstract hydrogen from the solvent to form the arene **2**. Whether the silver salt is a true catalyst or simply an initiator for a radical chain process has not been confirmed. The required temperature is slightly lower than those for other silver‐catalysed procedures, and *ortho* substituents are not a necessity in this system. Electron‐deficient substrates are preferred (e.g., **2a**–**c**) and methoxy‐substituted acids display poor reactivity (compounds **2t**, **2e**). Most impressive is the decarboxylation of unsubstituted benzoic acid to afford **2v**, which is unsuccessful in other procedures.

### 2.4. *ortho* Effect in Transition‐Metal‐Catalysed Decarboxylation

**Scheme 7:**
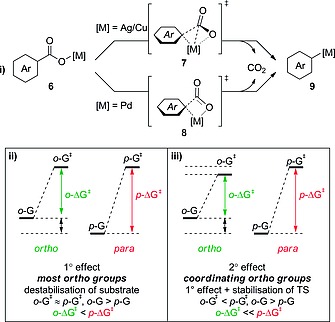
(i) General mechanism for the transition‐metal‐promoted decarboxylation of benzoic acid derivatives. (ii) Primary *ortho* effect (steric destabilisation). (iii) Secondary *ortho* effect (steric destabilisation + coordination).

Many groups have made efforts to understand the mechanism(s) of transition‐metal‐mediated decarboxylation.^22^ This has resulted in the general mechanism shown in Scheme 7. DFT studies suggest that the transition states for Ag‐ and Cu‐mediated decarboxylation are similar and involve the metal atom inserting into the aryl–carboxyl bond (intermediate **7**) with concomitant loss of CO_2_. The transition state for Pd‐mediated decarboxylation (intermediate **8**) is slightly different from that for Ag and Cu; in this case the carboxyl group is positioned perpendicular to the plane of the aryl group, rather than simply bent out of the plane. This results in a four‐membered transition state in which the palladium atom is bonded solely to the carboxyl oxygen atom, and there is no interaction with the carboxyl carbon atom.

In general, the transition‐metal‐mediated protodecarboxylation shows improved reactivity with *ortho*‐substituted benzoic acid derivatives. DFT studies have shown that the steric destabilisation imparted by an *ortho* substituent is the primary cause for this *ortho* effect. For example, the transition‐state energies of species bearing the same *ortho, meta* and *para* substituent are similar; however, the energy of the substrate bearing an *ortho* substituent is higher than that of the *meta* and *para* isomers. Overall this causes the barrier of decarboxylation to be lowered (Scheme 7, ii). This effect is apparent in all transition‐metal‐mediated procedures (Pd, Ag, Cu) and is in agreement with experimental results.

It has also been shown that, in the cases of copper and silver, some *ortho* substituents [e.g., NO_2_, C(O)H, C(O)NMe_2_] can lower the barrier to decarboxylation through coordination of the metal atom in the transition state. This causes a pronounced *ortho* effect, because the *ortho* group cannot only destabilise the substrate, but also stabilise the transition state, therefore leading to a greater decrease in the barrier to decarboxylation (Scheme 7, iii).

Finally, the electronic nature of the substituent also affects the decarboxylation barrier. For example, electron‐rich substituents are preferred for palladium‐mediated decarboxylation, whereas electron‐withdrawing substituents are preferred for copper‐ and silver‐mediated decarboxylation. DFT studies by Larrosa et al. revealed that, in the case of silver, electron‐withdrawing substituents lower the barrier to decarboxylation by minimising the build‐up of negative charge on the carbon atom *ipso* to the carboxyl group in the transition state.[22h] This stabilises the transition state and therefore lowers the barrier to decarboxylation. Because of the similarities between copper and silver decarboxylation a similar effect is likely in copper protodecarboxylation, though this has not been studied. No analogous DFT investigation into palladium protodecarboxylation has been performed; however, Hammett plot analysis suggests that a build‐up of positive charge occurs in the transition state in this case.[22f] This experimental study highlights the preference for electron‐donating substituents in palladium protodecarboxylation.

Overall, the factors that favour decarboxylation are threefold: (1) *ortho* substituents destabilise the starting benzoic acid/carboxylate through steric factors (Scheme 7, ii), (2) in some cases the *ortho* substituent can stabilise the transition state through coordination of the transition metal atom (Scheme 7, iii), and (3) depending on the metal employed, electron‐withdrawing or electron‐donating substituents stabilise the transition state by minimising the build‐up of negative/positive charge.

## 3. Decarboxylative C–CO_2_H/C–H Coupling

Coupling between benzoic acid derivatives and arenes is highly appealing, due to the ready availability of each set of substrates and the potential for developing atom‐economic procedures. Generally, these systems use palladium catalysts and stoichiometric silver salts as oxidants that, more often than not, also assist in the decarboxylation event. The couplings have been organised into sections on the basis of which transition metals are used in each procedure.

### 3.1. [Pd]/[Ag] Systems

**Scheme 8:**
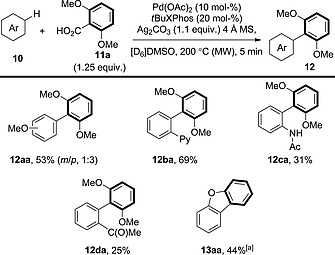
Scope of the decarboxylative oxidative coupling of 2,6‐dimethoxybenzoic acid with simple arenes. [a] From 2‐phenoxybenzoic acid.

The first examples of C–CO_2_H/C–H biaryl coupling were reported by Crabtree and co‐workers, who revealed that 2,6‐dimethoxybenzoic acid could be coupled with anisole to afford **12aa** or with arenes substituted with directing groups to give **12ba**–**12da** (Scheme 8).^10^ However, the yields of these reactions were generally low and led to the formation of significant amounts of protodecarboxylated products. The scope of the benzoic acid coupling partner was also not investigated, although they did report the intramolecular decarboxylative coupling of 2‐phenoxybenzoic acid to provide **13aa**.

**Scheme 9:**
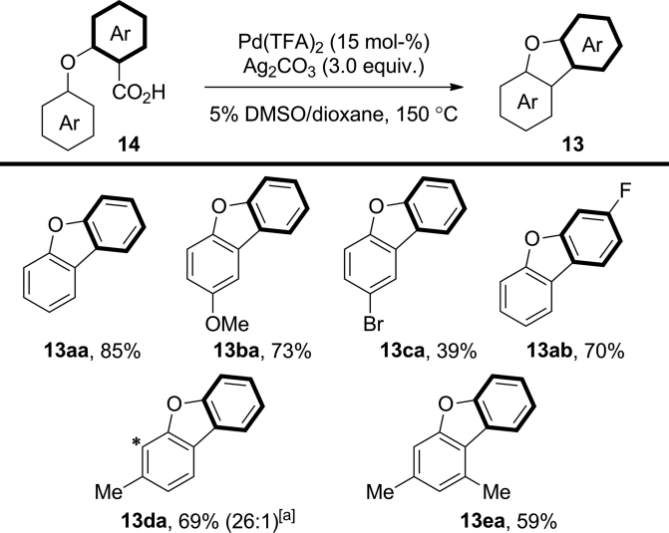
Scope of the intramolecular decarboxylative oxidative coupling of 2‐aryloxybenzoic acid derivatives. [a] Asterisk marks position of minor cyclised regioisomer.

Soon after, Glorius et al. reported an improved method for the decarboxylative cyclisation of 2‐aryloxybenzoic acid derivatives **14** (Scheme 9).^23^ This method provided better yields (85 % vs. 44 %) and lower levels of undesired protodecarboxylation (Ar–Ar/Ar–H = 1.0:1.2 vs. 1.0:0.01) for product **13aa** (Scheme 8 vs. Scheme 9). Electron‐rich and electron‐deficient substituents were tolerated on the C–H ring, but only one example of a substituent on the C–CO_2_H ring was reported (compound **13ab**). The cyclisation can occur at sterically hindered C–H bonds, though – if given the choice – the sterically less hindered C–H bond will undergo functionalisation preferentially (compounds **13da**, **13ea**).

**Scheme 10:**
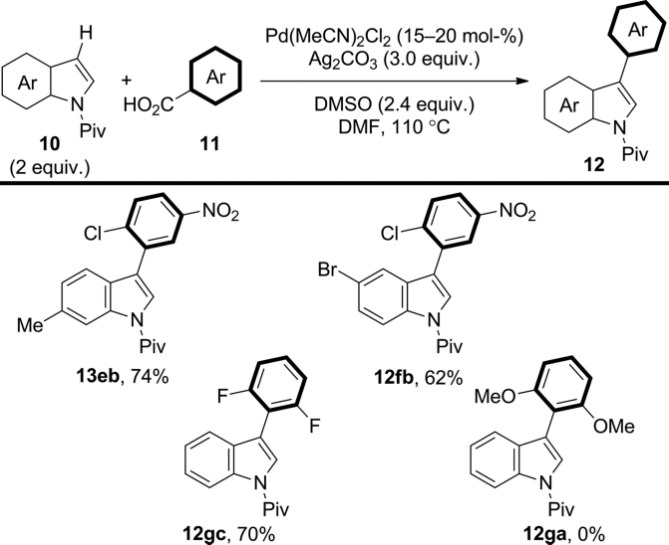
Scope of the decarboxylative oxidative coupling between indole derivatives and electron‐deficient benzoic acid derivatives.

Larrosa and co‐workers then looked to expand the cross‐coupling to incorporate electron‐deficient benzoic acid derivatives (Scheme 10).^24^ Under the optimised conditions, benzoic acid derivatives that are capable of undergoing silver‐mediated decarboxylation were reactive; however, 2,6‐dimethoxybenzoic acid was unreactive in this case and did not afford **12ga**. Also, the procedure was only applied to indole derivatives as the C–H coupling partners.^25^

**Scheme 11:**
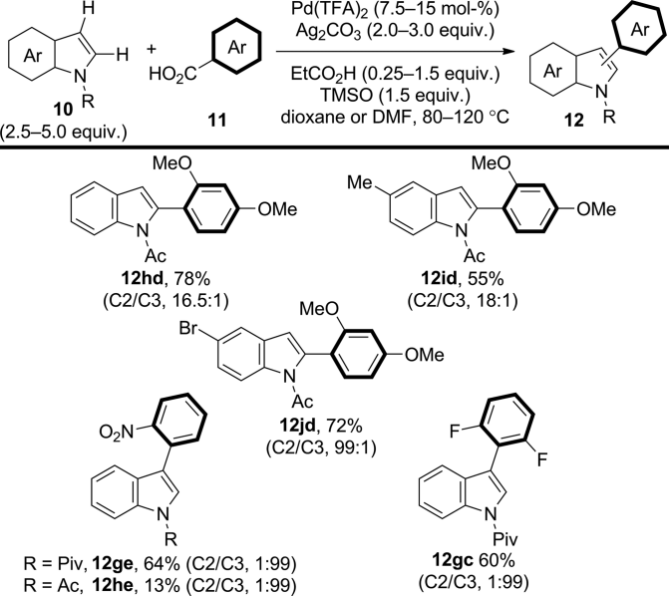
Scope of the decarboxylative oxidative coupling between indole derivatives and electron‐rich or electron‐deficient benzoic acid derivatives.

Su and co‐workers further optimised this methodology, with the resulting catalytic system being able to arylate both electron‐rich and electron‐deficient benzoic acid derivatives (Scheme 11).^26^ It seems that the choice of solvent (dioxane vs. DMF) and the addition of an aliphatic carboxylic acid (EtCO_2_H) were vital for obtaining reactivity with electron‐rich benzoic acid derivatives. Interestingly, the position (C2 vs. C3) of indole C–H arylation was determined solely by the electronic nature of the benzoic acid coupling partner: electron‐rich benzoic acid derivatives preferentially gave the C3‐arylated products, whereas electron‐deficient benzoic acid derivatives provided the C2 products, in accord with the Larrosa group's report. The reason for this switch in selectivity has not been investigated. Whereas acyl‐protected indole derivatives were preferred for coupling with electron‐rich benzoic acid derivatives, pivaloyl‐protected indole derivatives were required in order to obtain good reactivity with electron‐deficient coupling partners; however, the regioselectivity of the reaction was unaffected by the choice of protecting group (**12ge** vs. **12he**).

**Scheme 12:**
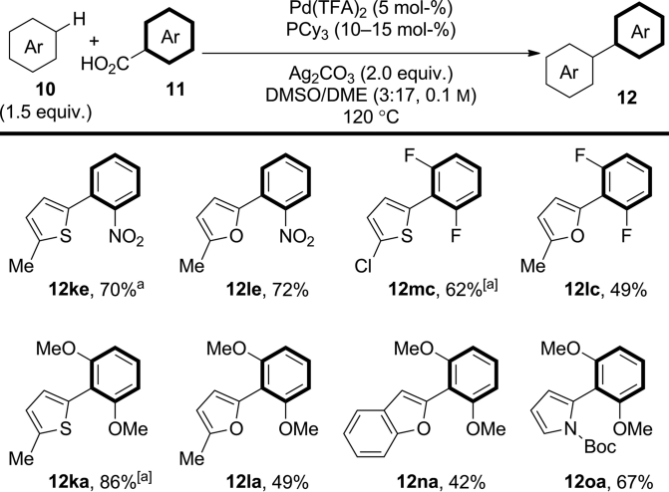
General schematic for the decarboxylative oxidative coupling between benzoic acid derivatives and heteroaromatics. [a] Ag_3_PO_4_ (1.5 equiv.), K_3_PO_4_ (2.0 equiv.).

Now that more general methods for the decarboxylative arylation of benzoic acid derivatives had been developed, studies then looked to expand the scope of the arene coupling partners. Continuing from their work on decarboxylative indole arylation, Su and co‐workers also reported that thiophene, furan, benzofuran and pyrrole derivatives could couple with a range of electron‐rich and electron‐deficient benzoic acid derivatives (Scheme 12).^27^

**Scheme 13:**
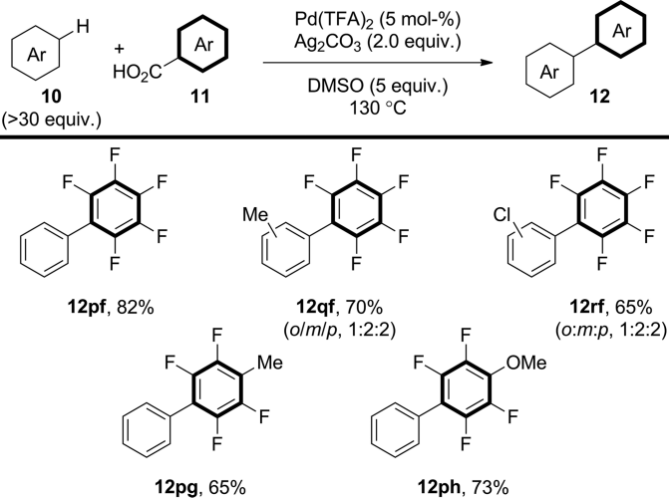
General schematic for the decarboxylative oxidative coupling between benzoic acid derivatives and simple arenes.

Luo et al. realised that simple arenes could also be used as coupling partners (Scheme 13).^28^ Unfortunately, the arene component was required in solvent quantities and the benzoic acid component was limited to highly activated polyfluorinated substrates. In addition, the selectivity of the C–H activation was poor. Nevertheless, this represents one of the few examples in which simple benzene has been used as a coupling partner, affording **12pf**, **12pg** and **12ph**. It is possible that the C–H activation occurs through an S_E_Ar‐type process; however, it is difficult to suggest this with confidence, because no detailed mechanistic studies have been performed.

**Scheme 14:**
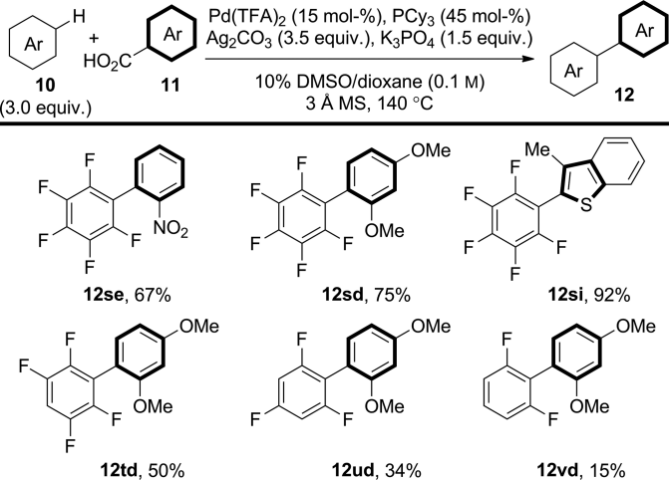
General schematic for the decarboxylative oxidative coupling between benzoic acid derivatives and polyfluorinated arenes.

Su and co‐workers reported coupling between polyfluorinated arenes and electron‐rich or electron‐deficient benzoic acid derivatives (Scheme 14). The reactivity of the arene coupling partner correlated with its acidity: thus, pentafluorobenzene was the most effective arene coupling partner, whereas 1,3‐difluorobenzene was poorly reactive.^29^ It is presumed that bis(arylation) is not observed in cases in which more than one acidic C–H bond is present (e.g., synthesis of **12td** or **12ud**); however, the authors do not comment on this possibility.

**Scheme 15:**
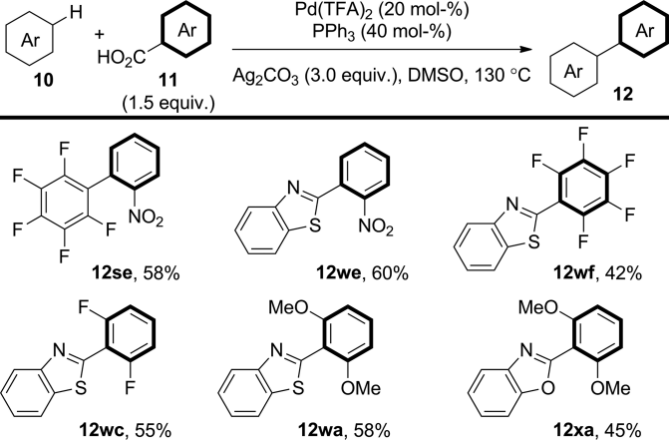
General schematic for the decarboxylative oxidative coupling between benzoic acid derivatives and arenes bearing acidic C–H bonds.

Similarly, Tan et al. also showed that polyfluoroarenes could couple with benzoic acid derivatives (Scheme 15).^30^ They also revealed that heteroarenes, such as benzoxazoles and benzothiazoles, could be used in this reaction with both electron‐rich and electron‐deficient benzoic acid derivatives.

**Scheme 16:**
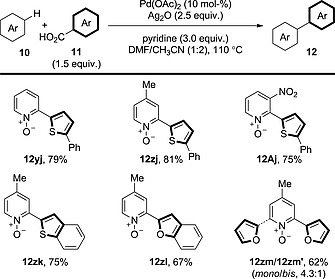
General schematic for the decarboxylative oxidative coupling of benzoic acid derivatives with arenes bearing acidic C–H bonds.

Another group of arenes that have proved to be reactive in C–H activation processes are pyridine *N*‐oxides. Muthusubrumanian and co‐workers employed these substrates in decarboxylative coupling with heteroaromatic carboxylic acid derivatives (Scheme 16).^31^ Unfortunately, the use of simple benzoic acid derivatives was not reported. In some cases, a significant amount of bis(arylated) product was observed resulting from two C–H activation events (e.g., **12zm′**).

### 3.2. [Pd]/[Cu] Systems

**Scheme 17:**
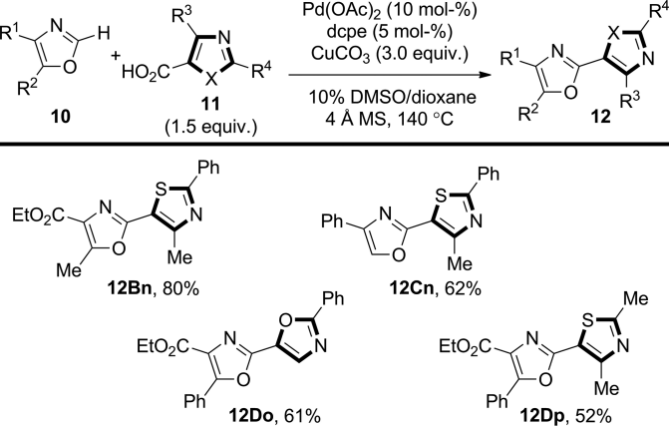
Scope of the decarboxylative oxidative coupling between azolecarboxylic acids and azoles in the presence of a [Pd]/[Cu] system.

Decarboxylative coupling between benzoic acid derivatives and aryl halides generally requires a system consisting of [Pd]/[Cu] salts;^3^ however, this type of system is hard to come by in oxidative couplings. In fact, the cross‐coupling between azolecarboxylic acid derivatives and simple azoles described by Greaney and co‐workers represents the only report in which a [Pd]/[Cu] dimetallic system is employed (Scheme 17).^32^ Although silver salts can be employed in this reaction, the authors preferred to use CuCO_3_ to aid the coupling of a range of substituted oxazoles with oxazole‐ and thiazolecarboxylic acid derivatives.

### 3.3. Cu‐Catalysed Procedures: [Cu]/[Ag] Systems and [Cu]‐Only Systems

**Scheme 18:**
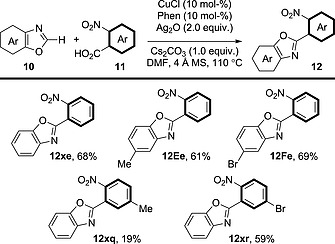
Scope of the decarboxylative oxidative coupling between 2‐nitrobenzoic acid derivatives and benzoxazole derivatives in the presence of a [Cu]/[Ag] system.

A [Cu]/[Ag] system analogous to the [Pd]/[Ag] system used in the coupling between benzoxazole derivatives and benzoic acid derivatives (Scheme 15) has been described (Scheme 18).^33^ The benzoxazole core can be substituted with electron‐donating or electron‐withdrawing groups at various positions; however, the procedure is limited to 2‐nitrobenzoic acid derivatives as coupling partners.

**Scheme 19:**
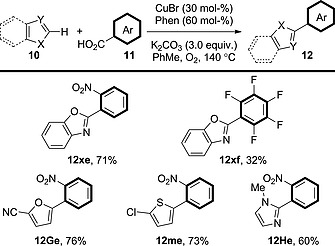
Scope of the decarboxylative oxidative coupling of heteroarenes in the presence of a [Cu]‐only system.

It was found that this coupling also proceeds on removal of the silver salt and its replacement with O_2_ as the sole oxidant (Scheme 19).^34^ Under these conditions, copper is the only transition metal present, and in catalytic quantities. The procedure shows a scope similar to – if not better than – that of the [Cu]/[Ag] system: polyfluorobenzoic acid derivatives can also be used in this procedure along with a wider range of heteroarenes such as thiophene, furan and imidazole derivatives. The use of only a catalytic quantity of an abundant transition metal is highly appealing; however, the procedure is only applicable to coupling between highly activated benzoic acid derivatives and heteroarenes. It would be of great interest if a more general procedure employing lower loadings of the catalyst were to be developed.

**Scheme 20:**
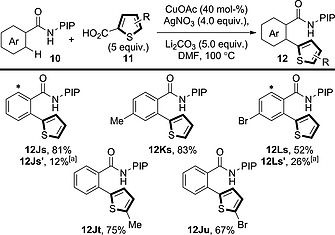
Scope of the directed decarboxylative oxidative coupling between benzamide derivatives and thiophenecarboxylic acid derivatives in the presence of a [Cu]/[Ag] system. PIP = (pyridin‐2‐yl)isopropyl. [a] Yield of bis(arylated) product. Asterisk marks the position of bis(arylation).

A [Cu]/[Ag] system has also been used for the decarboxylative arylation of benzamide derivatives through directed C–H functionalisation (Scheme 20).^35^ High loadings of CuOAc and super‐stoichiometric amounts of AgNO_3_ are required. The benzamide can be decorated at various points with electron‐donating or electron‐withdrawing groups; however, the authors only describe the decarboxylative coupling of thiophenecarboxylic acid derivatives. In some cases, such as those of **12Js** and **12Ls**, significant levels of bis(arylation) are observed.

### 3.4. [Ni]/[Ag] Systems

**Scheme 21:**
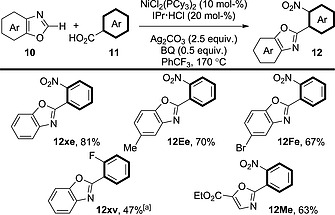
Scope of the decarboxylative oxidative coupling between 2‐nitrobenzoic acid derivatives and (benz)oxazole derivatives in the presence of an [Ni]/[Ag] system. [a] Ag_2_CO_3_ (3.0 equiv.); no BQ added.

We have already discussed the fact that [Pd]/[Ag], [Cu]/[Ag] and [Cu] alone (Schemes 15, 18 and 19) can promote decarboxylative coupling between benzoic acid derivatives and benzoxazole derivatives. In addition, an [Ni]/[Ag] procedure has been developed (Scheme 21).^36^ The scope is comparable with those of other procedures, showing good reactivity with electron‐deficient and electron‐rich benzoxazole derivatives and nitro/fluoro‐substituted benzoic acid derivatives.

### 3.5. [Ag]‐Only Systems

**Scheme 22:**
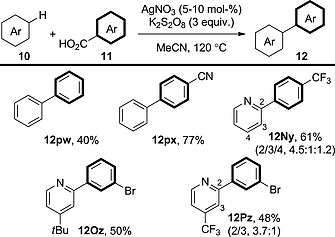
Scope of the decarboxylative oxidative coupling under radical conditions in the presence of an [Ag]‐only system.

The Minisci reaction – coupling between alkanecarboxylic acid derivatives and pyridine derivatives assisted by a silver catalyst – is well known; however, the extension of this procedure to incorporate aromatic carboxylic acids has been a longstanding challenge. Inspired by the Greaney group's report on the silver‐catalysed decarboxylation of benzoic acid derivatives under radical conditions (Scheme 5),^21^ Su and co‐workers used this methodology for an aromatic Minisci‐type reaction (Scheme 22).^37^ As mentioned in the discussion of the analogous protodecarboxylation, the beauty of this procedure is its ability to decarboxylate non‐*ortho*‐substituted benzoic acid derivatives. Because of the radical nature of the decarboxylation, a large excess (22.5 equiv.) of the arene is required, and the regioselectivity with respect to the C–H coupling partner is hard to control. The formation of biphenyl (**12pw**) is a nice addition to the reaction scope, because unsubstituted arenes and benzoic acid derivatives are poorly reactive in many C–H and decarboxylative arylations.

### 3.6. Summary

Coupling between *ortho*‐substituted benzoic acid derivatives and a variety of arenes has attracted increasing attention in recent years. The generality and economy of these procedures are slowly improving; however, there is still much room for investigation. For example, all the procedures, barring a report by Su and co‐workers (Scheme 22), are strictly limited to *ortho*‐substituted benzoic acid derivatives. The protodecarboxylation of non‐*ortho*‐substituted benzoic acid derivatives is possible (see Section 2), and coupling between non‐*ortho*‐substituted benzoic acid derivatives and aryl (pseudo)halides has also been intensely studied. Therefore, cross‐coupling of non‐*ortho*‐substituted benzoic acid derivatives is a realistic possibility. In terms of atom economy, decarboxylative oxidative couplings generally require the addition of stoichiometric amounts of transition metals (usually silver), and there are only two reports that do not require stoichiometric amounts of transition‐metal additives (Schemes 19 and 22). In the wider field of decarboxylative cross‐coupling, significant progress has been made in avoiding the need for stoichiometric amounts of transition metals, especially in coupling between benzoic acid derivatives and (pseudo)halides; therefore, it is hoped that similarly efficient procedures can be achieved for C–CO_2_H/C–H couplings.

## 4. Decarboxylative C–CO_2_H/C–CO_2_H Couplings

Coupling between benzoic acid derivatives and arenes through decarboxylation is highly appealing, because both reagent classes are readily available; however, because of the abundance of C–H bonds in the starting materials regioselectivity issues can be troublesome (cf. Schemes 13 and 22). Alternatively, if both coupling partners are benzoic acid derivatives the regioselectivity can be controlled by the position of the carboxyl group, whilst still achieving high atom economy. This field is still very much in its infancy, and only a handful of coupling procedures have been reported.

### 4.1. Homocoupling

**Scheme 23:**
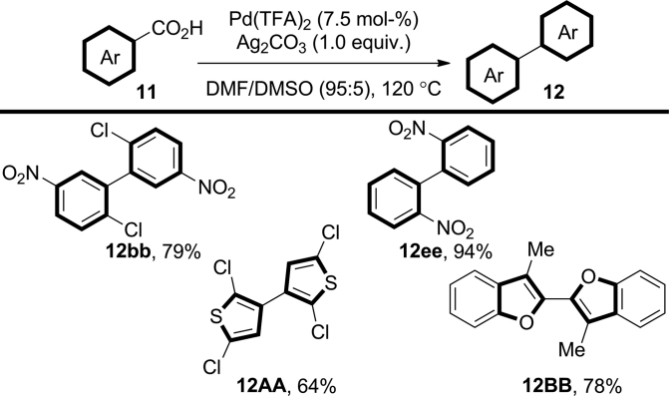
Scope of the double decarboxylative homocoupling of benzoic acid derivatives in the presence of a [Pd]/[Ag] system.

Reports in this field were begun by Larrosa et al., who performed homocoupling of (hetero)aromatic acid derivatives in the presence of a [Pd]/[Ag] system (Scheme 23).^11^ From examination of the reaction scope it is likely that silver is responsible for promoting the decarboxylation and that, upon transmetallation, palladium then can mediate the coupling of the aryl units.

**Scheme 24:**
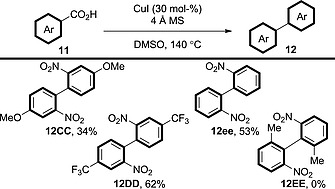
Scope of the double decarboxylative homocoupling of benzoic acid derivatives in the presence of a [Cu]‐only system.

More recently, a system for homocoupling catalysed solely by copper has been reported, but high loadings (30 mol‐%) of the copper catalyst are required (Scheme 24).^38^, ^39^ Remarkably, the coupling does not appear to require the addition of an oxidant, and it is performed under N_2_, so O_2_ is also excluded. Possibly DMSO is acting as the oxidant in this case; however, the authors do not comment on this peculiarity. The procedure is limited to *ortho*‐nitrobenzoic acid derivatives; sterically congested benzoic acid derivatives are unreactive (cf. **12EE**). Methods that can couple a wider range of benzoic acid derivatives in the presence of a similarly cost‐effective system would be highly interesting.

### 4.2. Cross‐Coupling

**Scheme 25:**
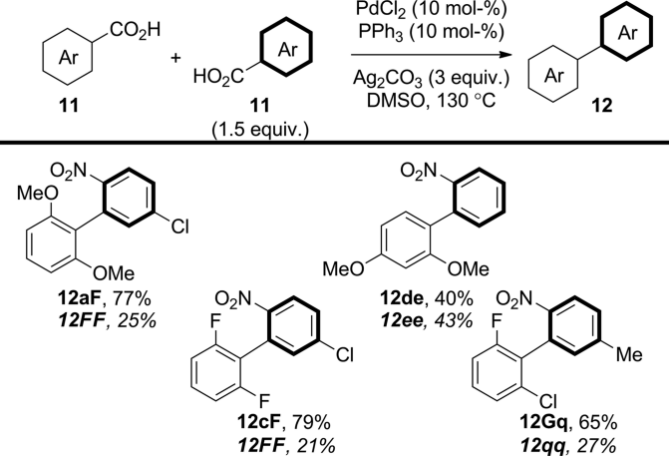
Scope of the double decarboxylative cross‐coupling of benzoic acid derivatives in the presence of a [Pd]/[Ag] system.

A much more valuable procedure would be the selective cross‐coupling of benzoic acid derivatives; however, differentiating between two benzoic acid components is a considerable challenge. The first attempt to tackle this problem was made by Tan, Deng and co‐workers, who found that electron‐rich or (poly)fluorinated benzoic acid derivatives can provide moderate to good yields in cross‐coupling with *ortho*‐nitrobenzoic acid derivatives (Scheme 25).^40^, ^41^ However, the chemoselectivity of this procedure is low, with at least 20 % of the homocoupled product of the 2‐nitrobenzoic acid (compound **12FF**, **12ee** or **12qq**) being observed in each case.

**Scheme 26:**
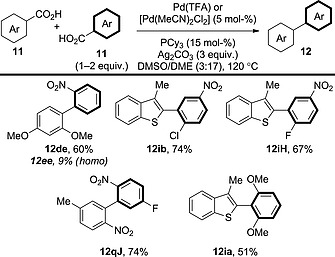
Scope of the double decarboxylative cross‐coupling of benzoic acid derivatives in the presence of a [Pd]/[Ag] system: an improved system.

Remarkably, by employing a different phosphine ligand and a slight adjustment of the solvent, Su et al. reported a similar system that could be applied to a wider range of benzoic acid derivatives, including benzoic acid derivatives that have similar electronic properties (Scheme 26).^42^ The chemoselectivity is certainly improved for product **12de** in comparison with the previous report; however, the cross‐coupling/homocoupling selectivity is still less than 7:1. Furthermore, the authors did not reveal the levels of homocoupling in each case, so the chemoselectivity of the procedure over a wide range of benzoic acid derivatives could not be assessed.

### 4.3. Summary

Selective coupling between two benzoic acid derivatives is an intriguing possibility. Although there are few reports in this area, they provide only a proof‐of‐principle for this transformation. Currently, these methods suffer from limitations similar to those described for cross‐coupling between benzoic acid derivatives and arenes. Namely, the procedures are limited to benzoic acid derivatives bearing *ortho* substituents, and the coupling of non‐*ortho*‐benzoic acid derivatives has not been reported. The need for the use of stoichiometric amounts of transition metals is also necessary in these procedures, and it is only in the homocoupling of *ortho*‐nitrobenzoic acid derivatives that catalytic amounts of copper can be used (Scheme 24). Finally, the levels of chemoselectivity for such cross‐couplings are very low, so strategies to allow better distinction between the benzoic acid derivatives are highly sought‐after. Despite these drawbacks, the initial reports in this area are beginning to display the potential of double decarboxylative couplings for biaryl synthesis.

## 5. Conclusions

Decarboxylative oxidative couplings are attractive procedures for three key reasons: (1) the carboxyl group offers a handle for selective couplings through decarboxylation, (2) the substrates are both inexpensive and readily available, and (3) they hold potential for the development of atom‐economic coupling procedures. This microreview describes the developments in this field from the early reports in 2008 to the present day.

The most intensively studied procedure has been the coupling between *ortho*‐substituted benzoic acid derivatives and a wide variety of arenes. A number of strategies have been adopted for this transformation, and it is hoped that future studies can continue to improve the generality and atom economy of these procedures.

Currently, there are only a handful of examples for the coupling of two benzoic acid derivatives. Many of the challenges associated with C–CO_2_H/C–H couplings are also apparent in C–CO_2_H/C–CO_2_H couplings, but with the added difficulty of distinguishing between the two benzoic acid derivatives in order to obtain good yields of cross‐coupled product. This point has been met with limited success, but it remains an important obstacle to be overcome in order to establish this strategy as a viable route for biaryl synthesis.

The field of decarboxylative oxidative cross‐coupling has developed significantly over the past decade. Current methods have already revealed some of the advantages of cross‐coupling of this type, and we are confident that future developments will continue to reveal the full potential of this reactivity.
